# Uncomplicated *Plasmodium falciparum* malaria in infants <5 kg: retrospective surveillance of hospital records in five Sub-Saharan African countries

**DOI:** 10.1186/1475-2875-11-S1-P4

**Published:** 2012-10-15

**Authors:** Maroufou J Alao, Adama D Gbadoè, Antoinette K Tshefu, Martin Meremikwu, Alfred B Tiono, Marc Cousin, Kamal Harried

**Affiliations:** 1 Service de Pédiatric, Hôpital de la Mère et de I’Enfant Lagune, 01 BP 107, Cotonou, Bénin; 2 Service de Pédiatric, CHU-Tokoin, BP 8881, Lomé, Togo; 3 Kinshasa School of Public Health, University of Kinshasa, BP 11850, Kinshasa, Democratic Republic of Congo; 4 Institute of Tropical Disease Research and Prevention, University of Calabar Teaching Hospital, GPO Box 1211, Calabar, Nigeria; 5 Centre National de Recherche et de Formation sur le Paludisme, Ministère de la Santé, Ouagadougou, Burkina Faso; 6 Novartis Pharma AG, CH-4002, Basel, Switzerland; 7 Novartis Pharmaceuticals Corporation, East Hanover, NJ 07936-1080, USA

## Background

WHO recommends artemisinin-based combination therapy (ACT) as first-line therapy for infants >5 kg of body weight (BW) with uncomplicated *P. falciparum* malaria. No ACTs are indicated in infants <4.5 kg. Coartem^®^ (20 mg artemether-120 mg lumefantrine, AL), with an available pediatric formulation, has a large clinical trial and postmarketing safety experience in infants ≥5 kg. Published reports on malaria in younger infants are scanty, leaving a significant knowledge gap about the pattern and outcome of malaria in this sub-population. The aim of the current study was to collect data on the clinical features, treatment and outcomes of uncomplicated *P. falciparum* malaria in infants <5kg in five countries within Sub-Saharan Africa.

## Materials and methods

Hospital charts ranging from 2006-2011 from eight hospitals in Bénin, Burkina Faso, the Democratic Republic of Congo, Nigeria, and Togo were retrospectively reviewed for inpatient and outpatient neonates and infants weighing <5 kg with parasitologically confirmed diagnosis of uncomplicated *P. falciparum* malaria. Details of clinical features, patient age group (<1 month or >1 month), treatment type received, and clinical outcomes were collected, and a descriptive analysis of data was carried out. A total of 907 cases were identified for inclusion.

## Results

The annual disease incidence ranged from 12 to 120 cases across hospitals and calendar years. There was a higher proportion of infants in the younger age group in Burkina Faso, Nigeria, and Togo, but in Bénin and the DRC the opposite was true. In all countries, the most frequent reason for seeking care was fever. Other reasons included dyspnea, cough or vomiting. Parasite density was generally low (<10% of the infants presented with parasitemia >5000/µL). In all countries other than Togo, quinine was the most common treatment (66% of patients overall). The second most common treatment overall was an ACT (22% of cases). The vast majority of patients recovered from their illness following treatment.

## Conclusions

Malaria in infants weighing <5 kg exists in Sub-Saharan Africa and calls for appropriate treatment. A prospective, multicenter, open-label study is currently planned to assess clinical efficacy, safety and pharmacokinetics of AL dispersible tablet in the treatment of acute uncomplicated *P. falciparum* malaria in infants <5 kg. Study results are expected in 2014.

